# Licorice: From Pseudohyperaldosteronism to Therapeutic Uses

**DOI:** 10.3389/fendo.2019.00484

**Published:** 2019-07-18

**Authors:** Chiara Sabbadin, Luciana Bordin, Gabriella Donà, Jacopo Manso, Giampiero Avruscio, Decio Armanini

**Affiliations:** ^1^Endocrinology Unit, Department of Medicine, University Hospital of Padua, Padua, Italy; ^2^Department of Molecular Medicine—Biological Chemistry, University of Padua, Padua, Italy; ^3^Angiology Unit, Department of Cardiac, Thoracic and Vascular Sciences, University Hospital of Padua, Padua, Italy

**Keywords:** licorice, aldosterone, polycystic ovary syndrome (PCOS), pseudohyperaldosteronism, glycyrrhetinic acid (GA), hypertension, inflammation

## Abstract

Licorice has been used as a medicinal plant from 2.500 years. It shows a wide range of biological and pharmacological activities, including anti-inflammatory and immune regulatory actions. One of its most known effects is the induction of hypertension, and it can induce what appears to be pseudohyperaldosteronism, due to glycyrrhetinic acid, the main active component of the root. Glycyrrhetinic acid and metabolites block the 11 beta-hydroxysteroid dehydrogenase type 2 and also bind mineralocorticoid receptors directly, acting as agonists. However, other interesting therapeutic uses of licorice are linked to its anti-androgen and estrogen-like activity, especially in the treatment of polycystic ovary syndrome (PCOS) in conjunction with spironolactone therapy. In this brief review, we report the main features and possible therapeutic uses of this ancient plant.

## Introduction

Licorice is a perennial plant, native to Mediterranean area and Asia Minor, studied for a long time as for its several biological and endocrine properties. The main constituents are triterpene saponins, whose main component is glycyrrhizic acid (GI) that is hydrolyzed into glycyrrhetinic acid (GA) in the stomach and duodenum. Other important constituents are flavonoids, such as glabridin, accounting about 1–24% of the root weight dependent on the species.

Licorice has been used as a medicinal plant for 2,500 years and its ancient applications were confirmed by recent scientific studies (1). In the fourth century B.C., Teophrastus reported that the Scythians were able to survive in the desert eating licorice roots as a thirst quencher and this effect was due to sodium and water retention, subsequently ascribed to the mineralocorticoid effect of licorice (2). Its most frequent side effect is pseudohyperaldosteronism, a clinical condition characterized by hypertension, hypokalemia and suppression of plasma renin and aldosterone levels (3). These effects were first reported by Revers in 1946 (4), and until 1970, licorice intoxication was a frequent cause of factitious hypertension and hypokalemia. Pseudohyperaldosteronism can be due to prolonged intake not only of licorice roots, but also of products flavored with licorice to ameliorate the taste, such as herbal products, candies, breath fresheners, tobacco, teas, and even laxatives (5). However, many other endogenous or exogenous conditions can lead to pseudohyperaldosteronism, such as Liddle's Syndrome, Cushing's syndrome, apparent mineralocorticoid excess (AME) syndrome, prolonged use of grapefruit (6), intake of fludrocortisone, and some enzyme deficiencies, such as deficiencies of 11 hydroxylase, 17 alpha-hydroxylase, or 5 alpha-reductase (7). The knowledge of the effects of overconsumption of mineralocorticoid-like substances has reduced the prevalence of this pathological condition.

**Figure 1:**
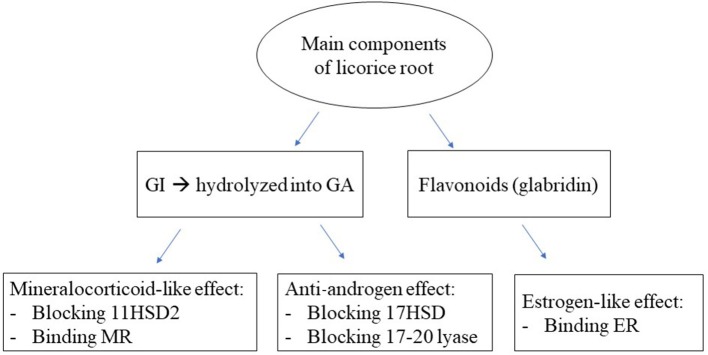
Endocrine effects of the main components of licorice. GI, glycyrrhizic acid; GA, glycyrrhetinic acid; 11HSD2, 11 beta-hydroxysteroid dehydrogenase 2; MR, mineralocorticoid receptor; 17HSD, 17 hydroxysteroid dehydrogenase; ER, estrogen receptor.

Moreover, licorice has several other clinical applications, due to its anti-androgen and estrogen-like activity (Figure 1), that are especially used in the treatment of polycystic ovary syndrome (PCOS) in association with spironolactone, a mineralocorticoid receptor (MR) blocker.

In this brief review, we report the features, mechanisms of endocrine actions and the possible therapeutic uses of licorice and its derivatives, focusing mainly on *in vitro* and *in vivo* studies in humans.

## The Mineralocorticoid Effect and the Mechanism of Pseudohyperaldosteronism

Aldosterone is the main mineralocorticoid, produced by the zona glomerulosa of the adrenal cortex, and it is mainly regulated by angiotensin II and potassium. It regulates electrolyte and water balance, and consequently blood pressure, by stimulating gene expression of serum-glucocorticoid-inducible kinase 1 (SGK1) and epithelial sodium channel (ENaC), involved in sodium and potassium regulation and reabsorption in renal tissues (8). Aldosterone exerts its effects via the MR, which belongs to the nuclear receptor superfamily and acts as a ligand-dependent transcription factor (9).

**Figure 2:**
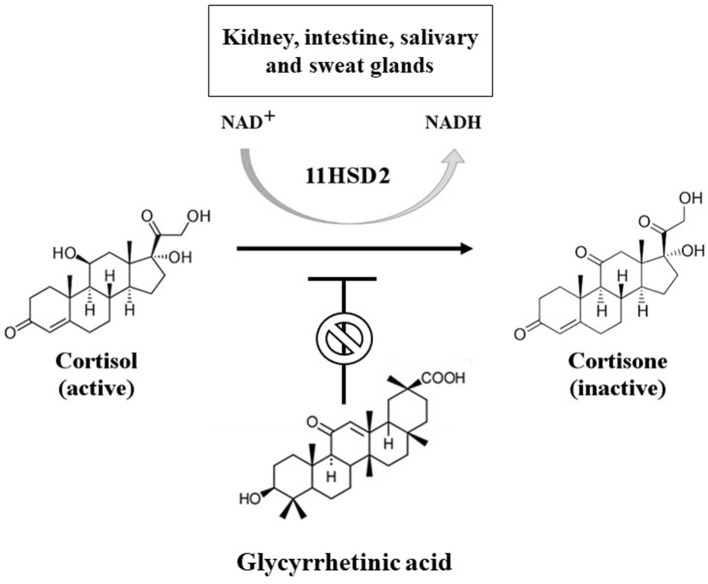
Mechanism of conversion of cortisol into cortisone through 11 beta-hydroxysteroid dehydrogenase 2 (11HSD2). This enzyme is a NAD-dependent dehydrogenase enzyme, mainly expressed in the kidney, intestine, salivary and sweat glands. It can be inhibited by glycyrrhetinic acid, leading to functional mineralocorticoid excess due to unmetabolized cortisol at the level of classical target tissues of aldosterone.

Cortisol, the main glucocorticoid, and aldosterone bind the MR with similar affinity, but cortisol levels are 100–1,000 times higher than that of aldosterone. For this reason, in the classical target tissues of aldosterone, such as kidney, intestine and salivary and sweat glands, the MR activation is regulated by the presence of the enzyme 11 beta-hydroxysteroid dehydrogenase type 2 (11HSD2), which inactivates cortisol to cortisone (Figure 2). Cortisone has no affinity for MR and glucocorticoid receptor, ensuring the activation of the MR only by aldosterone. However, if the enzyme is saturated (by significantly increased cortisol levels, such as in Cushing's syndrome) or mutated (as in AME syndrome) or blocked (as during prolonged intake of licorice), cortisol is able to bind and activate MR, leading to a pseudohyperaldosteronism (7).

Licorice-induced pseudohyperaldosteronism is mainly due to GA, which acts through two different mechanisms (Figure 1): it can block 11HSD2 (Figure 2) and directly bind to the MR as an agonist, with an affinity of about 5,000 times lower than aldosterone, evaluated in kidney cytosol or kidney slices from adrenalectomized rats (10). However, during chronic overconsumption of licorice, plasma concentrations of GA support a direct effect of GA at the MR level, notwithstanding the low affinity (11). Moreover, it has been demonstrated in human mononuclear leukocytes (MNL) by radioreceptor assay that the mineralocorticoid effector mechanism of GA can be reversed by coincubation with canrenone, a MR blocker, derivative of spironolactone (11). The effect on the 11HSD2 occurs even with relatively low serum concentrations of GA, while the binding to MR starts later when the substance has accumulated in the blood (12).

The accurate personal history, including a detailed dietary and drug history, and the measurement of renin, aldosterone and serum and urinary electrolytes are necessary for an accurate diagnosis of these forms of hypertension (13). The more accurate parameter to diagnose the reduced function of 11HSD2 is the measurement of the urinary cortisol metabolites, represented by the increased urinary cortisol/cortisone or urinary tetrahydrocortisol+allo-tetrahydrocortisol (TFH+alloTHF)/tetrahydrocortisone (THE) ratios (14). Increased urinary cortisol/cortisone ratio is an important tool, also for the differential diagnosis with Cushing's syndrome, since cortisol levels can be very high during chronic licorice intake (15).

The features of pseudohyperaldosteronism are linked to individual factors, such as the age, the gender, a previous history of hypertension, the time of transit and hydrolysis of GI to GA in the stomach and duodenum (10). Cessation of licorice intake and, in some cases, treatment with MR blockers can recover the abnormalities, but sometimes hypertension can persist due to other causes, as reported in some cases of primary aldosteronism after adrenalectomy.

## Mineralocorticoids, Licorice and Inflammation

In the last few decades, many studies have evaluated the role of aldosterone not only in the sodium balance and hypertension, but also in the pathogenesis of inflammation (16). This effect was hypothesized by Selye immediately after the discovery of the first mineralocorticoid, deoxycorticosterone (DOC). He reported an inflammatory activity of DOC in contrast to the anti-inflammatory action of glucocorticoids (17). The inflammatory effect of aldosterone is mediated through MR and non-genomic pathways not yet deeply clarified (16). Therefore, all the substances with a mineralocorticoid-like action may induce not only alterations of electrolytes and hypertension, but also inflammatory reactions in several tissues.

Since 1980, aldosterone excess has been reportedly involved in the inflammatory reaction, heart fibrosis, cardiovascular and cerebrovascular risk and atrial fibrillation. The protective effect of MR blockers, like spironolactone, canrenone, potassium canrenoate and eplerenone, to prevent the inflammatory effect of aldosterone in non-epithelial cells, was evidenced in the studies of Pitt and collaborators in patients with severe heart failure. The authors reported a significant reduction of the prevalence of heart failure relapse and cardio-cerebrovascular accidents when the MR blockers were added to the conventional treatments of these pathological conditions (18–20). The protective effect of MR blockers is opposite to the effect of other diuretics that increase aldosterone secretion and inflammatory reaction (21, 22). Recent researches are exploring the protective effect of spironolactone as an anti-inflammatory and immunomodulatory agent even in the treatment of other pathological conditions, such as rheumatoid arthritis and central serous chorioretinopathy (23, 24).

We investigated the inflammatory effect of aldosterone using MNL, which are the cells mainly involved in inflammation. In 1985, we characterized MR in MNL (25), and subsequently, we showed that *in vitro* aldosterone and GA were able to regulate the intracellular concentration of electrolytes (26) and the cellular volume (27), through both genomic and non-genomic pathways (28). In 2004 we investigated a possible involvement of aldosterone in the MNL model (29). Incubation of mononuclear cells with aldosterone enhanced the protein expression of PAI-1 and p22phox, two markers of inflammation. The effect of aldosterone was reversed by coincubation with canrenone. In the same study, we also evaluated the effect of GA, which was similar to aldosterone at concentrations consistent with its affinity for MR.

## Licorice as Anti-Inflammatory: the Erythrocyte Model

**Figure 3:**
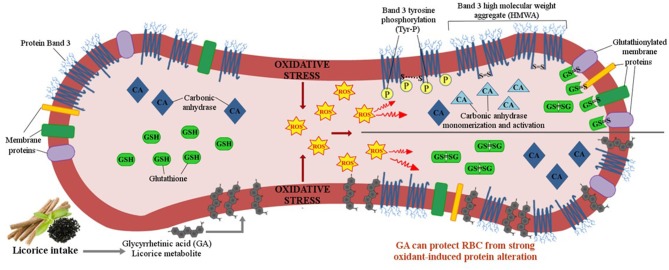
Red blood cell (RBC) membrane alterations induced by oxidative stress and the possible anti-inflammatory effect of licorice. Glycyrrhetinic acid (GA), which has been shown to directly interact with membrane sphingolipids and cholesterol, can induce a protective shield against membrane protein oxidation, mainly avoiding protein band 3, the main constituents of RBC membranes, from being oxidized by ROS to form high molecular weight aggregate (HMWA), recognized by circulating IgG as premature aged cells. GA also prevents protein glutathionylation, which would avoid cytosolic GSH store depletion and support the correct functioning of cytosolic enzymes such as Carbonic Anhydrase (CA2).

In erythrocytes, the proinflammatory effect has been evaluated by the increased sensitivity to diamide, a mild oxidant able to trigger the tyrosine phosphorylation (Tyr-P) of erythrocyte membrane proteins, mainly band 3 (30). The increased band 3 Tyr-P levels correlate with chronic deficiency of cellular anti-oxidative defenses. A previous study demonstrated that GA prevents the erythrocyte alterations induced by diamide in a dose-dependent manner, preserving membrane integrity against oxidative and proteolytic damage (31) (Figure 3). These effects mediated by GA are opposite from those of aldosterone, that exacerbates the alterations induced by diamide. The prooxidative effect of aldosterone was confirmed in both *in vitro* studies, incubating erythrocytes from healthy volunteers with increasing aldosterone concentrations, and *in vivo* experiments, evaluating erythrocytes from patients with primary aldosteronism (30). The erythrocyte membrane alterations were mediated by aldosterone in a dose- and time-dependent manner and were almost completely prevented by canrenone or cortisol, suggesting a non-genomic effect mediated by MR (32). Moreover, aldosterone-related alterations led to increased autologous IgG binding, which could induce premature erythrocyte removal from circulation (30).

The discrepancy between the effects of GA in MNL and erythrocytes demands an explanation. The protective effect could be related to a direct interaction of GA at the level of plasma membrane (30), not mediated by MR, preventing membrane protein oxidation (Figure 3) and/or to the glucocorticoid activity of GA (10). These data suggest that licorice can induce pseudohyperaldosteronism and an inflammatory reaction related to the binding to MR and to the blocking of 11HSD2, but it can also show anti-inflammatory and antiatherosclerotic (33) effects modulating cellular membrane fluidity and oxidative stress, and showing an estrogen-like (34) and glucocorticoid-like activity (10).

## Therapeutic Use of Licorice

Licorice root has been widely used for several therapeutic properties. In particular, GA has antiviral and antimicrobial activities, stimulating T-helper 1 immune response and the interferon gamma secretion (35). Licorice has also been used in the treatment of the chronic fatigue syndrome (36) and of Addison's disease, especially before the discovery of fludrocortisone (37): these effects are due to the reduced inactivation of cortisol in cortisone through the block of 11HSD2, even when cortisol production is reduced, and to the binding to the MR. These properties have been applied even in the treatment of postural hypotension in some diabetic patients with secondary hypoaldosteronism (38).

In recent years, the most fascinating use of licorice has been in the treatment of PCOS. We previously demonstrated that licorice can decrease the secretion of testosterone both in men and in women (39, 40). In healthy men this effect is not usually relevant, as the serum concentrations of testosterone are relatively high, while in women affected by hyperandrogenism, as in PCOS, the anti-androgen effect of licorice can be important, especially when associated with spironolactone. Spironolactone acts not only as MR blocker, but also as androgen receptor antagonist, affecting both gonadal and adrenal steroidogenesis and causing sex steroid-related clinical manifestations, such as impotence and gynecomastia in men, and menstrual irregularities and breast tenderness in women. Therefore, it is widely used in the treatment of PCOS, being effective for hirsutism, acne, effluvium capillorum and other clinical signs of hyperandrogenism (41). However, during treatment, patients may complain about side effects due to its anti-mineralocorticoid effect, causing hypotension and hyperkalemia. The addition of licorice can blunt the diuretic activity of spironolactone and enhance the anti-androgen effect (Figure 1), reducing androgen secretion by blocking the 17 hydroxysteroid dehydrogenase and 17–20 lyase in the ovaries and adrenals (42). It is worth noting that licorice can also reduce the prevalence on intermenstrual bleeding occurring in about 20–50% of women taking spironolactone (43). This effect could be related to the stimulation of aromatase activity and/or to the estrogen-like effect of glabridin, a flavonoid present in some commercial preparations of licorice (44). Recent studies support the estrogen-like effect of glabridin even at the level of bones in rats with ovariectomized-induced osteoporosis (45). All these properties of licorice can explain the use of the root even in the past as a remedy for sterility of women, reducing biochemical hyperandrogenism and improving ovulation rate (40), and probably as a sort of natural alternative to hormonal replacement therapy, improving symptoms of estrogen depletion (34). The effectiveness of herbal medicine containing licorice has been recently proven even in overweight PCOS patients (46). PCOS patients frequently have insulin-resistance, obesity and an increased risk of developing metabolic syndrome and cardiovascular disease, partially related to a proinflammatory systemic status (47) and a relative hyperaldosteronism (48) associated to this condition. These findings support the use of spironolactone in this syndrome and the cotreatment with licorice may improve even the metabolic alterations (49), blocking 11HSD1, and thus, reducing the availability of cortisol at the level of adipocytes. Indeed, in a previous study, we reported that the use of a cream containing 2.5% GA for 1 month significantly reduced the thickness of the superficial fat layer in comparison to a placebo cream, without changes in blood pressure, plasma renin, aldosterone and cortisol values (50).

In conclusion, licorice extracts have been used for more than 2,000 years as a medicinal plant and its consumption should not be limited by the possible onset of pseudohyperaldosteronism, since it can offer many other interesting therapeutic applications.

## Author Contributions

All authors listed have made a substantial, direct and intellectual contribution to the work, and approved it for publication.

### Conflict of Interest Statement

The authors declare that the research was conducted in the absence of any commercial or financial relationships that could be construed as a potential conflict of interest.

## References

[1] Armanini D Fiore C Mattarello MJ Bielenberg J Palermo M. History of the endocrine effects of licorice. Exp Clin Endocrinol Diabetes. (2002) 110:1–5. 10.1055/s-2002-34587 12373628

[2] Fiore C Eisenhut M Ragazzi E Zanchin G Armanini D. A history of the therapeutic use of liquorice in Europe. J Ethnopharmacol. (2005) 99:317–24. 10.1016/j.jep.2005.04.015 15978760 PMC7125727

[3] Conn JW Rovner DR Cohen EL Licorice-induced pseudoaldosteronism. Hypertension, hypokalemia, aldosteronism, and suppressed plasma renin activity. JAMA. (1968) 205:492–6. 10.1001/jama.205.7.492 5695305

[4] Revers FE Heedt succus liquiritiae een gegenzde werking of de maagweer? Nedl Tijdschr Geneesk. (1946) 90:135–7. 20291620

[5] Scali M Pratesi C Zennaro MC Zampollo V Armanini D Pseudohyperaldosteronism from liquorice-containing laxative. J Endocrinol Invest. (1990) 13:847–8. 10.1007/BF03349639 2096161

[6] Palermo M Armanini D Delitala G. Grapefruit juice inhibits 11beta-hydroxysteroid dehydrogenase *in vivo*, in man. Clin Endocrinol. (2003) 59:143–4. 10.1046/j.1365-2265.2003.01806.x 12807517

[7] Sabbadin C Armanini D. Syndromes that mimic an excess of mineralocorticoids. High Blood Press Cardiovasc Prev. (2016) 23:231–5. 10.1007/s40292-016-0160-5 27251484

[8] Booth RE Johnson JP Stockand JD. Aldosterone. Adv Physiol Educ. (2002) 26:8–20. 10.1152/advan.00051.2001 11850323

[9] Fuller PJ Yao Y Yang J Young MJ. Mechanisms of ligand specificity of the mineralocorticoid receptor. J Endocrinol. (2012) 213:15–24. 10.1530/JOE-11-0372 22159507

[10] Armanini D Karbowiak I Funder JW Affinity of licorice derivatives for mineralocorticoid and glucocorticoid receptors. Clin Endocrinol. (1983) 19:606–12. 10.1111/j.1365-2265.1983.tb00038.x 6315264

[11] Armanini D Wehling M Weber PC Mineralocorticoid effector mechanism of licorice derivates in human mononuclear leukocytes. J Endocrinol Invest. (1989) 12:303–6. 10.1007/BF03349993 2549114

[12] Armanini D Lewicka S Pratesi C Scali M Zennaro MC Zovato S. Further studies on the mechanism of the mineralocorticoid action of licorice in humans. J Endocrinol Invest. (1996) 19:624–9. 10.1007/BF03349029 8957748

[13] Sontia B Mooney J Gaudet L Touyz RM. Pseudohyperaldosteronism, liquorice, and hypertension. J Clin Hypertens. (2008) 10:153–7. 10.1111/j.1751-7176.2008.07470.x 18256580 PMC8109973

[14] White PC Mune T Agarwal AK. 11 beta-Hydroxysteroid dehydrogenase and the syndrome of apparent mineralocorticoid excess. Endocr Rev. (1997) 18:135–56. 10.1210/edrv.18.1.0288 9034789

[15] Ceccato F Trementino L Barbot M Antonelli G Plebani M Denaro L. Diagnostic accuracy of increased urinary cortisol/cortisone ratio to differentiate ACTH-dependent Cushing's syndrome. Clin Endocrinol. (2017) 87:500–7. 10.1111/cen.13391 28590513

[16] Sabbadin C Calò LA Armanini D. The story of spironolactones from 1957 to now: from sodium balance to inflammation. G Ital Nefrol. (2016) 33.S66.12. 26913880

[17] Selye H. Stress and inflammation. Am J Proctol. (1953) 4:229–30. 13080458

[18] Pitt B Zannad F Remme W Cody R Castaigne A Perez A. The effect of spironolactone on morbidity and mortality in patients with severe heart failure. Randomized Aldactone Evaluation Study Investigators. N Engl J Med. (1999) 341:709–17. 10.1056/NEJM199909023411001 10471456

[19] Pitt B Remme W Zannad F Neaton J Martinez F Roniker B. Eplerenone, a selective aldosterone blocker, in patients with left ventricular dysfunction after myocardial infarction. N Engl J Med. (2003) 348:1309–21. 10.1056/NEJMoa030207 12668699

[20] Armanini D Sabbadin C Donà G Bordin L Clari G. Aldosterone receptor blockers spironolactone and canrenone: two multivalent drugs. Expert Opin Pharmacother. (2014) 15:909–12. 10.1517/14656566.2014.896901 24617854

[21] Ma J Albornoz F Yu C Byrne DW Vaughan DE Brown NJ. Differing effects of mineralocorticoid receptor-dependent and -independent potassium-sparing diuretics on fibrinolytic balance. Hypertension. (2005) 46:313–20. 10.1161/01.HYP.0000174327.53863.86 15998706

[22] Armanini D Bordin L Clari G. Serum potassium, thiazides, aldosterone, and mineralocorticoid receptors. Hypertension. (2012) 60:e9. 10.1161/HYPERTENSIONAHA.112.197889 22733466

[23] Singh H Bala S Jain D Jagota R Mathur R. Spironolactone (an adjuvant therapy) in rheumatoid arthritis: a case control study. Reumatologia. (2018) 56:87–91. 10.5114/reum.2018.75519 29853723 PMC5974630

[24] Wang SK Sun P Tandias RM Seto BK Arroyo JG. Mineralocorticoid receptor antagonists in central serous chorioretinopathy: a meta-analysis of randomized controlled trials. Ophthalmol Retina. (2019) 3:154–60. 10.1016/j.oret.2018.09.003 31014765

[25] Armanini D Strasser T Weber PC. Characterization of aldosterone binding sites in circulating human mononuclear leukocytes. Am J Physiol. (1985) 248(3 Pt1):E388–90. 10.1152/ajpendo.1985.248.3.E388 3156509

[26] Wehling M Armanini D Strasser T Weber PC. Effect of aldosterone on sodium and potassium concentrations in human mononuclear leukocytes. Am J Physiol. (1987) 252(4 Pt 1):E505–8. 10.1152/ajpendo.1987.252.4.E505 2952017

[27] Wehling M Kuhls S Armanini D. Volume regulation of human lymphocytes by aldosterone in isotonic media. Am J Physiol. (1989) 257(2 Pt 1):E170–4. 10.1152/ajpendo.1989.257.2.E170 2764099

[28] Wehling M. Specific, nongenomic actions of steroid hormones. Annu Rev Physiol. (1997) 59:365–93. 10.1146/annurev.physiol.59.1.365 9074769

[29] Calò LA Zaghetto F Pagnin E Davis PA De Mozzi P Sartorato P. Effect of aldosterone and glycyrrhetinic acid on the protein expression of PAI-1 and p22(phox) in human mononuclear leukocytes. J Clin Endocrinol Metab. (2004) 89:1973–6. 10.1210/jc.2003-031545 15070972

[30] Bordin L Donà G Sabbadin C Ragazzi E Andrisani A Ambrosini G. Human red blood cells alterations in primary aldosteronism. J Clin Endocrinol Metab. (2013) 98:249–501. 10.1210/jc.2012-3571 23539731

[31] Fiore C Bordin L Pellati D Armanini D Clari G. Effect of glycyrrhetinic acid on membrane band 3 in human erythrocytes. Arch Biochem Biophys. (2008) 479:46–51. 10.1016/j.abb.2008.08.011 18778682

[32] Bordin L Saccardi C Donà G Sabbadin C Andrisani A Ambrosini G. Mineralocorticoid receptor is involved in the aldosterone pathway in human red blood cells. Am J Transl Res. (2016) 8:314–28. 27158328 PMC4846885

[33] Fogelman Y Gaitini D Carmeli E. Antiatherosclerotic effects of licorice extract supplementation on hypercholesterolemic patients: decreased CIMT, reduced plasma lipid levels, and decreased blood pressure. Food Nutr Res. (2016) 60:30830. 10.3402/fnr.v60.30830 27113136 PMC4845696

[34] Boonmuen N Gong P Ali Z Chittiboyina AG Khan I Doerge DR. Licorice root components in dietary supplements are selective estrogen receptor modulators with a spectrum of estrogenic and anti-estrogenic activities. Steroids. (2016) 105:42–9. 10.1016/j.steroids.2015.11.006 26631549 PMC4714869

[35] Fiore C Eisenhut M Krausse R Ragazzi E Pellati D Armanini D. Antiviral effects of Glycyrrhiza species. Phytother Res. (2008) 22:141–8. 10.1002/ptr.2295 17886224 PMC7167979

[36] Baschetti R Chronic fatigue syndrome and liquorice. N Z Med J. (1995) 108:156–7. 7761058

[37] Borst JG Ten Holt SP De Vries LA Molhuysen JA. Synergistic action of liquorice and cortisone in Addison's and Simmonds's disease. Lancet. (1953) 1:657–63. 10.1016/S0140-6736(53)91800-5 13036119

[38] Basso A Dalla Paola L Erle G Boscaro M Armanini D. Licorice ameliorates postural hypotension caused by diabetic autonomic neuropathy. Diabetes Care. (1994) 17:1356. 10.2337/diacare.17.11.1356 7821181

[39] Armanini D Bonanni G Palermo M. Reduction of serum testosterone in men by licorice. N Engl J Med. (1999) 341:1158 10.1056/NEJM199910073411515 10515764

[40] Armanini D Mattarello MJ Fiore C Bonanni G Scaroni C Sartorato P Palermo M. Licorice reduces serum testosterone in healthy women. Steroids. (2004) 69:763–6. 10.1016/j.steroids.2004.09.005 15579328

[41] Armanini D Andrisani A Bordin L Sabbadin C. Spironolactone in the treatment of polycystic ovary syndrome. Expert Opin Pharmacother. (2016) 17:1713–5. 10.1080/14656566.2016.1215430 27450358

[42] Armanini D Castello R Scaroni C Bonanni G Faccini G Pellati D. Treatment of polycystic ovary syndrome with spironolactone and licorice. Eur J Obstet Gynecol Reprod. (2007) 131:61–7. 10.1016/j.ejogrb.2006.10.013 17113210

[43] Sabbadin C Andrisani A Zermiani M Donà G Bordin L Ragazzi E. Spironolactone and intermenstrual bleeding in polycystic ovary syndrome with normal BMI. J Endocrinol Invest. (2016) 39:1015–21. 10.1007/s40618-016-0466-0 27072668

[44] Sakamoto K Wakabayashi K. Inhibitory effect of glycyrrhetinic acid on testosterone production in rat gonads. Endocrinol Jpn. (1988) 35:333–42. 10.1507/endocrj1954.35.333 2850159

[45] Klasik-Ciszewska S Kaczmarczyk-Sedlak I Wojnar W. Effect of glabridin and glycyrrhizic acid on histomorphometric parameters of bones in ovariectomized rats. Acta Pol Pharm. (2016) 73:517–27. 27180445

[46] Arentz S Smith CA Abbott J Fahey P Cheema BS Bensoussan A. Combined lifestyle and herbal medicine in overweight women with Polycystic Ovary Syndrome (PCOS): a randomized controlled trial. Phytother Res. (2017) 31:1330–40. 10.1002/ptr.5858 28685911 PMC5599989

[47] Donà G Sabbadin C Fiore C Bragadin M Giorgino FL Ragazzi E Inositol administration reduced oxidative stress in erythrocytes of patients with polycystic ovary syndrome. Eur J Endocrinol. (2012) 166:703–10. 10.1530/EJE-11-0840 22223702

[48] Armanini D Bordin L Donà G Sabbadin C Bakdounes L Ragazzi E. Polycystic ovary syndrome: implications of measurement of plasma aldosterone, renin activity and progesterone. Steroids. (2012) 77:655–8. 10.1016/j.steroids.2012.02.010 22387621

[49] Sil R Ray D Chakraborti AS. Glycyrrhizin ameliorates insulin resistance, hyperglycemia, dyslipidemia and oxidative stress in fructose-induced metabolic syndrome-X in rat model. Indian J Exp Biol. (2013) 51:129–38. 23923606

[50] Armanini D Nacamulli D Francini-Pesenti F Battagin G Ragazzi E Fiore C. Glycyrrhetinic acid, the active principle of licorice, can reduce the thickness of subcutaneous thigh fat through topical application. Steroids. (2005) 70:538–42. 10.1016/j.steroids.2005.01.007 15894038

